# A multi-modal profiling workflow of transcriptomics and biomineralization demonstrated on a tooth-on-chip model

**DOI:** 10.1016/j.mex.2026.104137

**Published:** 2026-08-31

**Authors:** Chong Huang, Chloë Tijhof, Wei Ji, Fang Yang, X. Frank Walboomers

**Affiliations:** a Department of Dentistry – Regenerative Biomaterials, Research Institute for Medical Innovation, Radboud university medical center, Philips van Leijdenlaan 25, Nijmegen, 6525 EX, the Netherlands; b The State Key Laboratory Breeding Base of Basic Science of Stomatology (Hubei-MOST) & Key Laboratory of Oral Biomedicine Ministry of Education, School & Hospital of Stomatology, Wuhan University, Wuhan, China; c Department of Implantology, School & Hospital of Stomatology, Wuhan University, Wuhan, China

**Keywords:** Tooth-on-chip, Organ-on-chip, Fibrin hydrogel, Magnetic cell separation, Spatial transcriptomics, Mineralization

## Abstract

tooth-on-chip platforms recapitulate dental epithelial–mesenchymal (DE–DM) interactions, offering physiologically relevant in vitro models for tooth regeneration. However, broader adoption requires chip-scale analytical methods capable of resolving cell-type-specific transcriptional programs and characterizing mineral formation. Here, we present a multi-modal workflow adapting five established approaches to fibrin hydrogel-based tooth-on-chip constructs. Whole-construct RNA extraction yielded high-integrity RNA suitable for bulk RNA sequencing, enabling pooled transcriptional comparisons between dental epithelium and mesenchyme. Magnetic-activated cell sorting achieved efficient recovery and enrichment from chip-relevant cell inputs, while translating this approach to intact constructs revealed a platform-level incompatibility between fibrin dissolution and downstream sorting. Fixation strategies preserved tissue morphology for spatial transcriptomics but came at the cost of RNA quality, and Raman spectroscopy combined with transmission electron microscopy enabled ultrastructural assessment of calcium phosphate deposition, though confirming mature hydroxyapatite required further validation. Together, this workflow establishes a practical framework for benchmarking analytical readouts in fibrin-based tooth-on-chip and related organ-on-chip co-culture systems.

Established whole-construct RNA extraction for bulk transcriptional profiling of DE-DM co-cultures

Adapted cell sorting and dissociation strategies for cell-type-specific enrichment, identifying key compatibility constraints

Applied spatial transcriptomics preparation and spectroscopic/ultrastructural imaging to characterize tissue architecture and calcium phosphate deposition

## Graphical abstract

**Figure:**
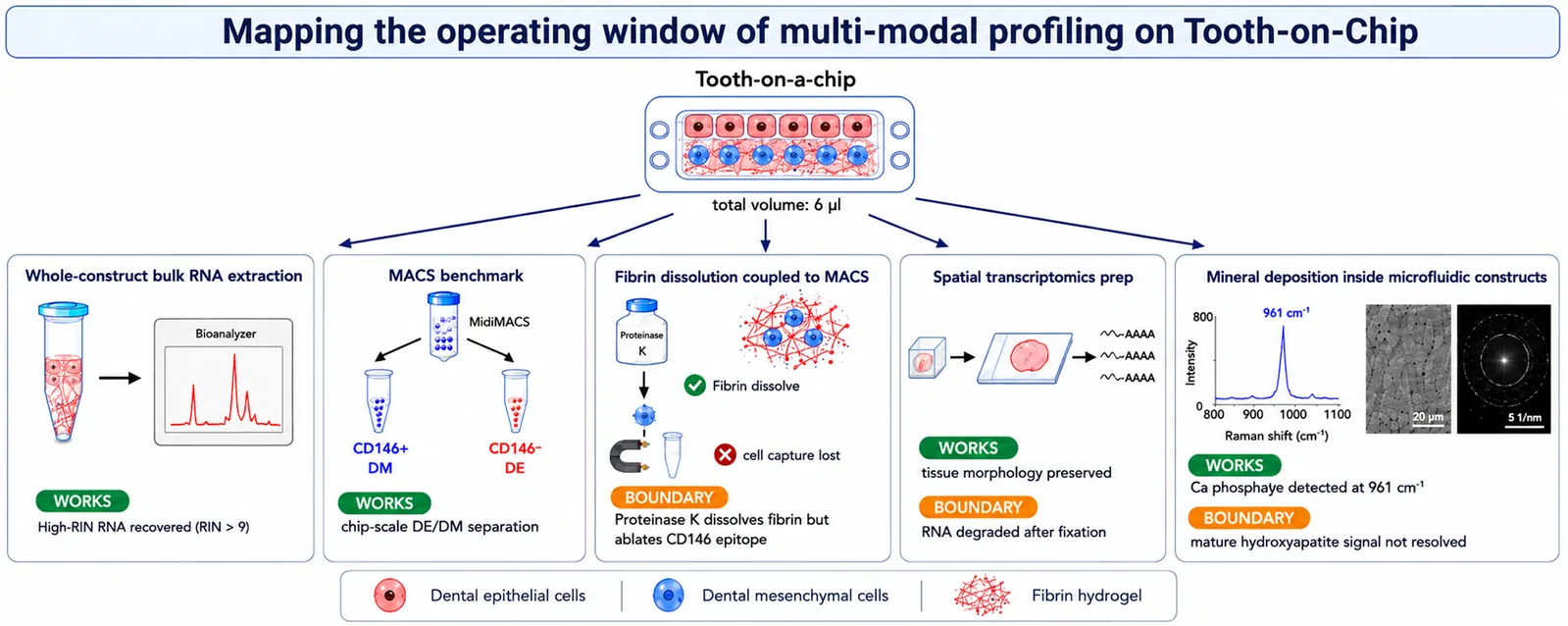

## Specifications table

**Table:** 

**Subject area**	Medicine and Dentistry
**More specific subject area**	Organ-on-chip; dental tissue engineering; multi-modal profiling of 3D co-cultures
**Name of your method**	Multi-modal profiling workflow for tooth-on-chip
**Name and reference of original method**	N/A
**Resource availability**	All reagents, instruments and software are listed in the Method details section. No bespoke code or data are used.

## Background

The tooth is one of the most highly mineralized structures in the human body, and rebuilding its development in vitro remains a central challenge in regenerative dentistry. Over the past decade, tooth regeneration models have progressed from conventional two-dimensional cultures to organoids, spheroids, engineered three-dimensional constructs, and emerging tooth-on-chip platforms [[1], [2], [3], [4], [5]]. Recent tooth-on-chip models have progressed beyond simplified well-plate cultures by recreating defined dental tissue structures within controlled microenvironments [6]. This progress has expanded the scope of tooth-on-chip research from tissue-specific response testing to the study of more complex multicellular and developmental processes [7,8]. For example, early studies mainly used dentin–pulp interfaces to investigate biomaterial, biofilm, and therapeutic responses[9,10], whereas newer systems increasingly incorporate stem-cell niches [11], epithelial–mesenchymal interactions [12], vascular components, and innervation [3]. As model complexity increases, analytical characterization becomes more demanding. Accordingly, current Tooth-on-chip studies still rely largely on model-specific cellular and tissue-level readouts, while the adaptation of more advanced molecular and physicochemical analyses to chip-scale constructs remains comparatively underexplored [8].

These analytical limitations motivated the workflow developed in the present study. In our previous work, the tooth-on-chip platform successfully recapitulated the dental epithelial (DE) –dental mesenchymal (DM) interface during tooth development and responded faithfully to defined growth-factor stimulation [12]. However, broader application of this platform requires analytical methods capable of characterizing the model at greater biological depth. Specifically, two key features remain difficult to resolve: i) cell-type-resolved transcriptomic read-outs capturing the dynamic, heterogeneous fate decisions that drive cell differentiation [[13], [14], [15]], and ii) direct physicochemical characterization of the mineral phase, which in most dental in vitro studies is still inferred from calcium-binding histological stains (e.g., Alizarin Red S) and a small panel of mineralization biomarkers [16,17]. These analytical gaps are amplified by the chip format itself: the small culture volume limits cell yield, the fibrin matrix complicates biomolecule extraction and in situ signal detection. Therefore, workflows established for conventional plate cultures or bulk three-dimensional constructs cannot be assumed to translate directly to chip-scale co-cultures.

This article therefore describes a systematic, multi-modal analytical workflow that adapts five established techniques to intact tooth-on-chip constructs. For the transcriptomic pipeline, RNA quality was first assessed to determine whether bulk sequencing was technically viable. Cell dissociation strategies and magnetic-activated cell sorting (MACS) -based DE/DM enrichment were then tested to evaluate the feasibility of cell-type-resolved sequencing. The upstream processing steps for spatial transcriptomics on the Xenium platform were also assessed. For mineralization characterization, Raman spectroscopy and transmission electron microscopy (TEM) were applied to probe calcium phosphate deposition and ultrastructure within 21-day constructs. For each technique, the protocol, chip-scale feasibility, technical limitations, and fit-for-purpose boundaries are defined. The workflow therefore would potentially also be transferable to other fibrin-based organ-on-chip co-cultures.

## Method details

### Cell culture and tooth-on-chip fabrication

#### Cell culture

Cell isolation, maintenance, and expansion were performed as described in our previous study [12]. Briefly, dental epithelial (DE) cells were cultured in LHC-8 medium (Gibco, Thermo Fisher Scientific, Waltham, MA, USA) . Dental mesenchymal (DM) cells were cultured in alpha minimum essential medium (α-MEM; Gibco, Thermo Fisher Scientific) supplemented with 10% fetal bovine serum (FBS; Gibco,) and 1% penicillin/streptomycin (Gibco) . All cells were maintained at 37 °C in a humidified incubator containing 5% CO₂. DM cells between passages 3 and 6 were used for all experiments.

#### Tooth-on-chip fabrication and co-culture

The tooth-on-chip platform was fabricated and operated as described in our previous study [12]. In brief, DM cells were embedded in fibrin gel prepared from 10 mg/mL fibrinogen (Sigma-Aldrich Merck KGaA, Darmstadt, Germany) and loaded into the central channel of the chip. After 3 days of DM pre-culture, DE cells were introduced through the side channel to establish a three-dimensional (3D) DE–DM interface within the chip. Constructs were cultured under differentiation conditions containing 10 nM dexamethasone, 10 mM β-glycerophosphate, and 50 µg/mL vitamin C (all from Sigma-Aldrich) for 14 or 21 days, depending on the experiment.

### Workflow arm 1 — whole-construct bulk RNA extraction and quality assessment

**Fig. 1:**
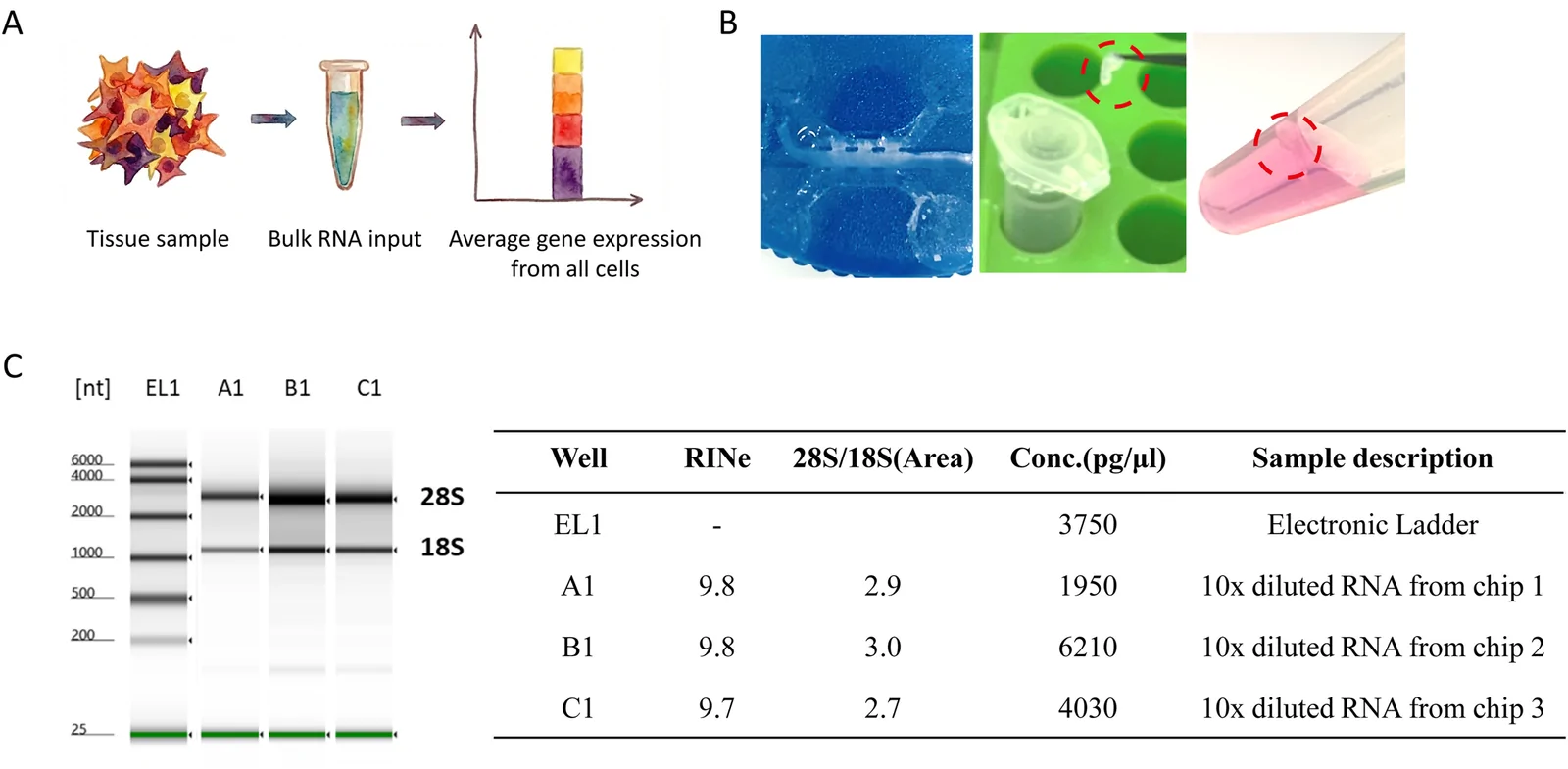
Whole-construct RNA quality from the tooth-on-chip supports bulk transcriptomic analysis. **(**A) Schematic illustrating the principle of bulk RNA sequencing: total RNA is extracted from an intact tissue sample, capturing the average gene expression profile across all constituent cell types. (B) Photographs of construct retrieval from the tooth-on-chip. Left: PDMS layer removed to expose the cell-laden fibrin construct. Middle: the intact construct collected from the central channel (red dashed circle) . Right: the recovered construct immersed in TRIzol reagent for RNA isolation (red dashed circle) . (C) RNA integrity and quantity assessment. Left: Agilent 2100 Bioanalyzer electropherogram of RNA from three independent chip replicates (A1, B1, C1) together with the electronic ladder (EL1) , which provides size-reference fragments. Each sample lane shows two prominent rRNA peaks corresponding to 28S (∼4000 nt) and 18S (∼2000 nt) , whose sharp and well-defined profiles indicate high RNA integrity. The green 25-nt band present in all lanes represents the internal marker used for alignment and quantification. Right: summary of RNA quality metrics, including RINe values (9.7–9.8) , 28S/18S area ratios (2.7–3.0) , and RNA concentrations measured at 10 × dilution (1950–6210 pg/µL).

**Rationale*.*** Bulk RNA sequencing enables genome-wide differential expression analysis and pathway-level interpretation of growth-factor responses (Fig. 1A) . The first analytical question is therefore whether the chip yields enough intact RNA to enter a standard library preparation.

#### Protocol

**RNA extraction.** After 14 days of co-culture, the entire cell-laden fibrin construct was retrieved from each chip, rinsed in phosphate-buffered saline (PBS; Gibco) , and immersed in 200 µL TRIzol Reagent (Invitrogen, Thermo Fisher Scientific) . The construct was mechanically disrupted, adjusted to a final volume of 1 mL with additional TRIzol Reagent, and incubated at room temperature for 15 min. Chloroform (Sigma-Aldrich) was then added at 200 µL per sample. Samples were shaken vigorously, allowed to phase-separate, and centrifuged at 4 °C for 15 min. The aqueous phase was transferred to an RNeasy Micro Kit column (QIAGEN, Hilden, Germany) and purified according to the manufacturer’s instructions, including on-column DNase treatment to remove residual genomic DNARNA eluates were diluted 10-fold prior to quality control.

**RNA quality assessment.** RNA concentration and integrity were assessed on an Agilent 2100 Bioanalyzer using the RNA 6000 Nano Kit (Agilent Technologies, Santa Clara, CA, USA) . The RNA assay chip was prepared with gel-dye mix per according to the manufacturer instructions. Samples were denatured at 70 °C for 2 min, placed on ice, and 1 µL was loaded per well. The RNA assay chip was vortexed for 1 min and run on the Bioanalyzer for automated electrophoresis and fluorescence detection. RNA Integrity Number equivalent (RINe) values and 28S/18S area ratios were recorded.

### Workflow arm 2 — DE/DM enrichment by magnetic cell separation

**Rationale*.*** Bulk RNA from intact chips reflects the average expression of DE and DM populations and cannot resolve cell-type-specific responses. Physical separation prior to library preparation is therefore needed. To identify a sorting strategy compatible with chip-scale cell yields, we compared two magnetic-activated cell sorting (MACS) systems with different separation formats across the same input range.

#### Protocol

• **MACS systems and labelling**. Two MACS systems were compared for separating DE and DM populations: i.e. (1) the MagnaRack™ (Thermo Fisher) and (2) the MidiMACS™ Separator (Miltenyi Biotec, Bergisch Gladbach, Germany) . For these optimization experiments, DE and DM cells were obtained from conventional 2D cultures rather than recovered from intact tooth-on-chip constructs. In both systems, cells were labeled with anti-CD146 magnetic microbeads (Miltenyi Biotec) to capture the CD146-positive DM fraction. The unlabeled flow-through was collected as the DE-enriched population. MagnaRack™ enables column-free magnetic pelleting for small samples, whereas MidiMACS™ uses column-based high-gradient separation to achieve higher purity.
• **Cell Recovery Rate.** To evaluate recovery efficiency, defined numbers of DM cells (10⁷, 10⁶, and 10⁵) were dissociated into single-cell suspensions and subjected to MACS. Cell pellets were resuspended in 60 µL MACS buffer supplemented with 20 µL FcR blocking reagent and 20 µL anti-CD146 MicroBeads, and incubated for 15 min at 4 °C. Reagent volumes were kept constant across all input cell numbers. After labeling, cells were washed and processed by magnetic separation to obtain CD146-positive and CD146-negative fractions. Recovered cells from both fractions were counted immediately, and post-sort recovery was calculated as a percentage of the initial input.
• **Morphological validation of enrichment.** To assess enrichment under controlled mixed-cell conditions, DE and DM cells cultured separately in 2D were combined at a defined 1:1 ratio to generate total input populations of 10⁷, 10⁶, or 10⁵ cells. The mixtures were labelled with anti-CD146 magnetic MicroBeads and separated using the MidiMACS™ system. The CD146-negative DE-enriched flow-through and CD146-positive DM-enriched retained fractions were collected separately and seeded into 24-well plates at 3, 000 cells/cm². Brightfield images were acquired 24 h after seeding. For 10⁶ and 10⁵ group, three replicate wells were analyzed, with five regions assessed per well. Epithelial-like cells were identified by cobblestone morphology and manually counted by two independent observers. The epithelial-like fraction was calculated as epithelial-like cells/total cells. Observer measurements were averaged for each region and then across the five regions to obtain one value per well.

### Workflow arm 3 — fibrin dissolution coupled to MACS

**Rationale.** To perform MACS on chip-derived constructs, the cells must first be released from the fibrin matrix. Three criteria must be met simultaneously: dissolution of the complete gel; preservation of the surface epitope (s) used for MACS; and maintenance of cell viability. An enzymatic and a non-enzymatic option were tested against these three criteria.

#### Protocol

• **Fibrin Dissolution.** For enzymatic digestion, proteinase K (Qiagen) solutions were prepared in d-PBS at concentrations of 2, 0.2, and 0.02 mg/mL. Fibrin gels (10 μL, 10 mg/mL) without cells were incubated with 300 μL of proteinase K solution at 37 °C, and gel dissolution was documented photographically at 25 and 90 min. A no-enzyme control (Dulbecco’s phosphate-buffered saline (D-PBS, Gibco) only) was included. For non-enzymatic dissolution, two urea formulations were tested: (1) 5 M urea (Sigma-Aldrich) in demineralized water and (2) 5 M urea in 50 mM Tris/150 mM NaCl (Sigma-Aldrich) . Fibrin gels (10 mg/mL) were incubated in each solution at 37 °C, and dissolution was documented after 24 h.
• **Viability and surface-marker integrity downstream of dissolution.** To assess whether proteinase K was compatible with downstream MACS, DM cells (1 × 10⁶) harvested from a 2D culture flask were treated with proteinase K (2 mg/mL, 37 °C) or left untreated as a control. Cells were first labeled with anti-CD146 microbeads and subjected to MidiMACS separation. After the separation step, cell viability was determined by trypan blue exclusion (Sigma-Aldrich) . An aliquot of the cell suspension was mixed 1:1 with 0.4 % trypan blue, and live (unstained) and dead (blue) cells were counted using a hemocytometer. Live cell recovery was expressed as a percentage of total input. The MACS capture fraction of CD146+ cells was calculated as labeled cells in the positive fraction divided by total input.

### Workflow arm 4 — upstream sample preparation for spatial transcriptomics

**Rationale*.*** Spatial transcriptomics maps gene expression to specific locations within intact tissues (Fig. 4A) and is the most direct way to recover cell-type-resolved expression on the DE–DM interface without dissociating the construct. The key methodological question was whether the standard upstream preparation steps for spatial transcriptomics, including fixation, optimal cutting temperature compound embedding, cryo-sectioning, hematoxylin and eosin staining, and destaining, were compatible with soft fibrin-based chip construct.

#### Protocol

**Fixation and embedding.** After 14 days of co-culture, the samples were washed once with PBS at room temperature (RT) and immersed in 4% formalin for 20 min at RT. Samples were washed three times with PBS (5 min each, RT) and briefly immersed in 1% eosin (2 min) to aid visualization during embedding. Constructs were transferred into Optimal Cutting Temperature (OCT) compound in a cryomold dimensioned for the 6.5 × 6.5 mm capture area. Four samples were aligned within a single reading box. Two additional samples were embedded in OCT in Eppendorf tubes as matched RNA quality controls. All samples were snap-frozen in isopentane (VWR International, Leuven, Belgium) on dry ice and cryosectioned at 10 µm.

**Hematoxylin and eosin (H&E) and Destaining.** H&E staining was performed to assess preservation of cellular morphology and tissue architecture. Cryosections were stained with Delafield hematoxylin (Sigma-Aldrich) for 8 min, washed in running tap water for 10 min, dehydrated through graded ethanol, counterstained with eosin for 2 min, further dehydrated (90% ethanol, 1 min; 100% ethanol, 2 min) , cleared in 1:1 xylene:ethanol followed by two xylene baths (5 min each) , and mounted with dibutylphthalate polystyrene xylene (DPX) medium (Sigma-Aldrich) . Slides were dried overnight before imaging.

### Workflow arm 5 — raman spectroscopy and TEM of in situ mineralization

**Rationale.** Most dental in vitro studies rely on Alizarin Red S, which does not distinguish hydroxyapatite from amorphous calcium phosphate or dystrophic mineral. To extract compositional and structural information on the calcium phosphate deposition directly within the chip, confocal Raman was coupled with TEM and selected-area electron diffraction (SAED) .

#### Protocol

**Raman spectroscopy.** To assess the chemical composition and spatial distribution of calcium phosphate deposition within DE-DM interface, Raman spectroscopy was performed on tooth-on-chip samples after 21 days of co-culture. Constructs were fixed in 4% formalin for 1 h at room temperature. Brightfield imaging was first used to identify regions of interest at the DE–DM interface, which were subsequently subjected to confocal Raman analysis. Raman measurements were conducted using a WITec alpha300R Raman microscope (Oxford Instruments WITec GmbH, Ulm, Germany) ) equipped with a 63×/1.2 W water-immersion objective (Zeiss W C-Apochromat, Carl Zeiss AG, Oberkochen, Germany) . Raman maps were acquired over a 28 × 28 µm area with a lateral step size of 1 µm, using 457 nm laser excitation (15 mW at the sample) and a pixel dwell time of 6 s. Spectra were collected in the range of 400–3000 cm⁻¹ with a spectral resolution of 2–3 cm⁻¹. Acquired datasets were analyzed using WITec Project SIX software (v6.1) , and spectral components were resolved by multivariate analysis and mapped to their corresponding spatial locations.

**TEM.** To examine the ultrastructure and crystallinity of mineral deposits within the constructs, TEM (Talos F100C G2, Thermo Fisher Scientific) was performed on tooth-on-chip samples after 21 days of co-culture. Constructs were fixed in 4% formalin for 1 h at room temperature and air-dried for 24 h. Dried samples were prepared as bulk fragments, gently smeared onto copper TEM grids coated with a thin carbon film. Ultrastructural imaging and selected area electron diffraction (SAED) were carried out at an accelerating voltage of 200 kV. In SAED, the electron beam is diffracted by the crystal lattice of the specimen, producing a diffraction pattern that reveals the crystal structure and phase identity of the mineral deposits. These patterns were analyzed to assess the crystal structure and degree of mineral ordering within the deposited phase.

### Statistical analysis

Quantitative data are presented as mean ± standard deviation. Two-group comparisons were performed using unpaired Student's *t*-tests. Significance levels: *p < 0.05, **p < 0.01, ***p < 0.001.

## Method validation

### Whole-construct RNA integrity and quantity supports bulk transcriptomic analysis

Bulk RNA sequencing enables genome-wide differential expression analysis and pathway-level interpretation of growth-factor responses (Fig. 1.A) . To assess RNA recovery from the tooth-on-chip system, total RNA was extracted from intact chip constructs and evaluated for yield and integrity (Fig. 1.B) . All samples demonstrated high RNA integrity: RIN^e^ values were 9.8, 9.8, and 9.7 for Chip 1 (A1) , Chip 2 (B1) , and Chip 3 (C1) respectively. Sharp 18S and 28S ribosomal RNA bands were visible on the electropherogram with no sign of degradation (Fig. 1.C) . The 28S/18S area ratios ranged from 2.7 to 3.0, confirming predominance of intact 28S rRNA—a hallmark of non-degraded eukaryotic RNA.

RNA concentrations at 10 × dilution ranged from 1.95 to 6.21 ng/µL (Fig. 1C) . With an elution volume of 14 µL, the estimated total yield per chip was 273–869 ng. This exceeds the 100 ng minimum required by standard bulk RNA-seq library preparation kits (e.g., Illumina TruSeq Stranded mRNA) . Together, the high RIN^e^ values (≫7.0 threshold) and sufficient yields confirmed the feasibility of whole-construct transcriptomic analysis on the tooth-on-chip platform.

### MidiMACS outperforms MagnaRack for DE/DM separation

**Fig. 2:**
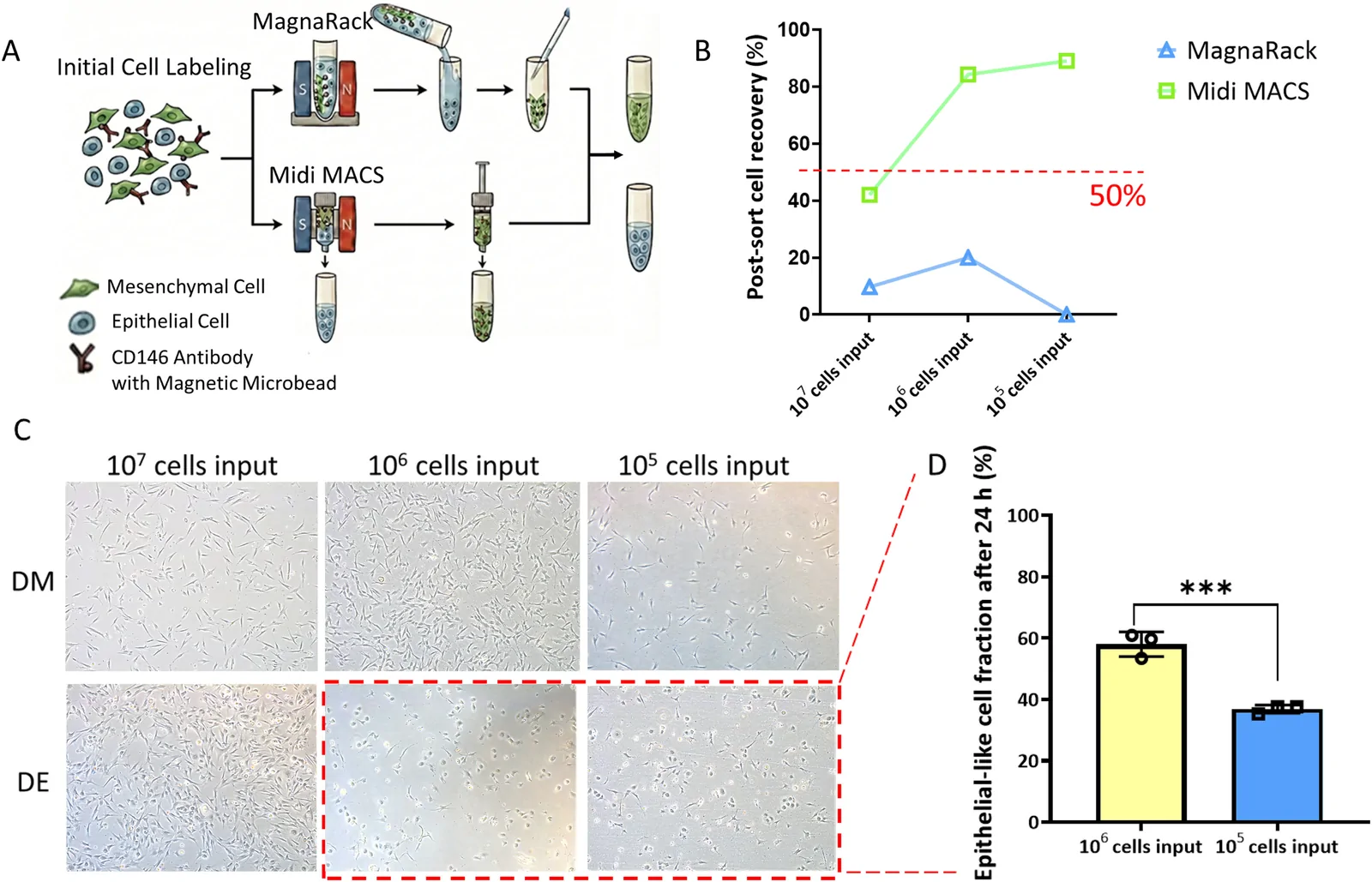
MidiMACS outperforms MagnaRack for magnetic separation of DE and DM populations. (A) Schematic of the two magnetic separation workflows. After initial labelling with anti-CD146 magnetic microbeads, cells are processed through either the MagnaRack or the MidiMACS system. The MagnaRack is a static tube-based magnet that retains labelled cells against the tube wall. The MidiMACS is a high-gradient column-based separator in which labelled cells are retained within a ferromagnetic matrix. In both systems, the CD146⁺ DM fraction is magnetically retained, while the unlabelled flow-through is collected as the DE-enriched fraction. (B) Comparison of post-sort cell recovery between MagnaRack and MidiMACS at different input cell numbers. Recovery is expressed as the percentage of total input cells recovered across both fractions after separation (10⁷, 10⁶, 10⁵) . Blue triangles indicate MagnaRack and green squares indicate MidiMACS. The dashed red line marks the 50% recovery threshold. (C) Representative brightfield images of sorted fractions after MidiMACS separation at 10⁶ cell input (DE:DM 1:1) . Cells were seeded at 3, 000 cells/cm² and imaged after 24 h.DM fractions (top row) exhibit predominantly spindle-shaped mesenchymal morphology. DE fractions (bottom row) contain mixed populations of cobblestone-shaped epithelial cells and spindle-shaped mesenchymal contaminants. Quantification of epithelial-like cells in DE fractions at 10⁶ and 10⁵ inputs (red dashed box) is shown in (D) . Scale bar: 200 µm. (D) Quantification of epithelial-like cell fraction (%) in the DE fraction at 10^6^ and 10^5^ inputs. Data are presented as mean ± SD (n = 3) . ***p < 0.001, unpaired Student’s *t*-test.

Reliable separation of DE and DM populations after co-culture is essential for cell-type–specific molecular analysis. Two magnetic separation systems were compared across a range of input cell numbers (Fig. 2A) .

At 10^7^ cells input, both systems showed low recovery: MidiMACS below 50% and MagnaRack below 20%, likely reflecting column overloading or bead saturation. As cell input decreased, MidiMACS recovery improved markedly—reaching approximately 80% at both 10^6^ and 10^5^ inputs, whereases MagnaRack remained near 20% at 10^6^ and fell to near zero at 10^5^ (Fig. 2B) . Using 50% as a practical benchmark, MidiMACS consistently exceeded this threshold at lower cell inputs (10^6^ and 10^5^ cells amount) , making it more suitable for the limited cell yields of individual tooth-on-chip constructs.

To validate enrichment quality, cells from each MidiMACS fraction were seeded and imaged after 24 h (Fig. 2C) . DM fractions showed predominantly spindle-shaped mesenchymal morphology across all inputs. DE fractions contained a mixture of cobblestone-shaped epithelial cells and spindle-shaped contaminants. At the 10^7^ input, the DE fraction contained a conspicuous excess of DM-like cells compared with the 10^6^ and 10^5^ groups, further indicating that column overloading compromises separation quality.

Given the superior recovery of MidiMACS at lower inputs, the enrichment purity was quantified at 10^6^ and 10^5^ (Fig. 2D) . At 10^6^ input, approximately 55–65 % of cells in the DE fraction displayed epithelial-like morphology, compared with 30–40 % at 10^5^ input (p < 0.001) . Thus, MidiMACS achieves meaningful DE enrichment at moderate inputs, but purity remained input-dependent with varying degrees of residual non-target cell carryover.

### Proteinase K enables fibrin dissolution but compromises surface marker integrity

**Fig. 3:**
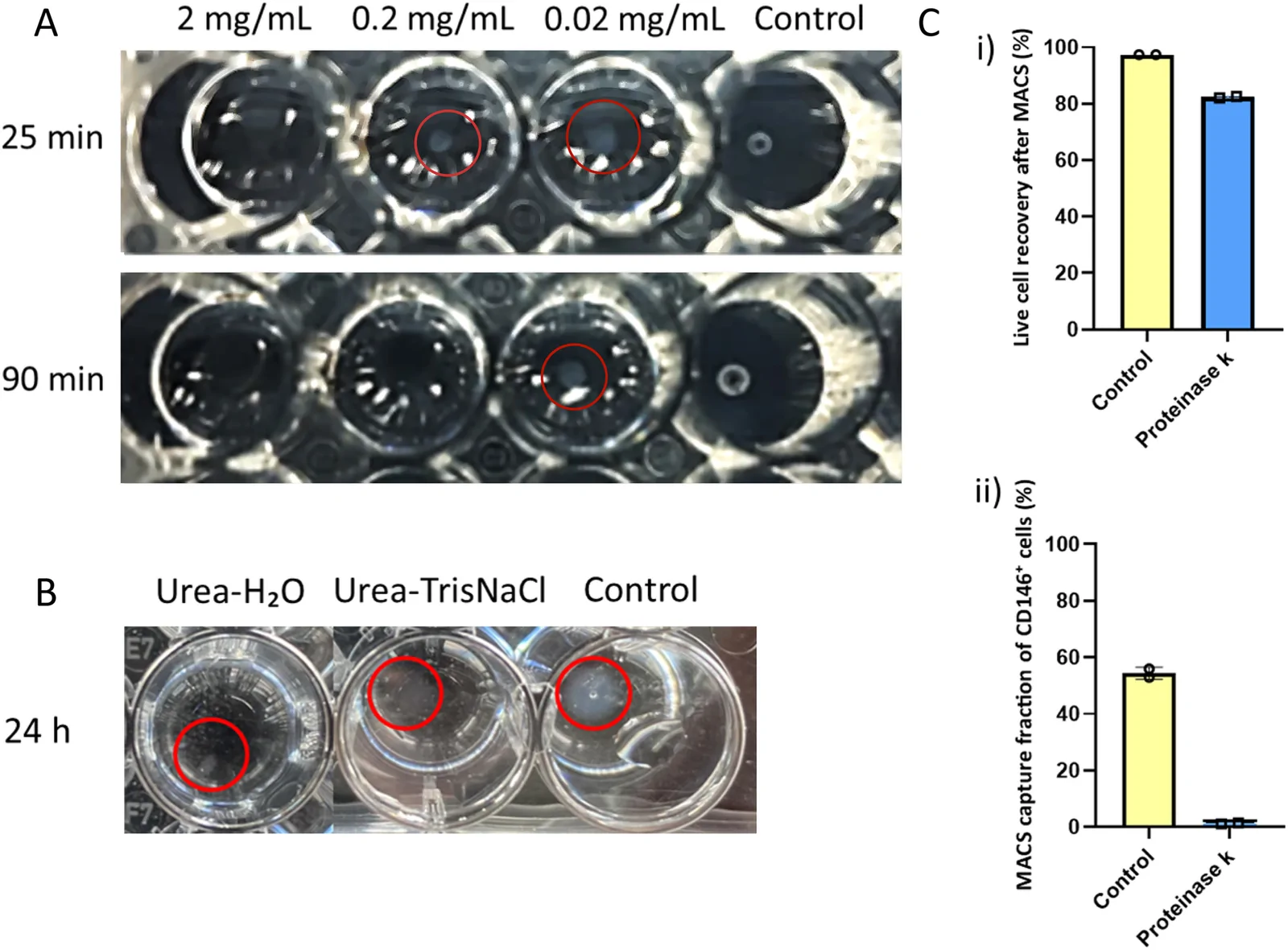
Proteinase K dissolves fibrin gels but destroys the CD146 epitope required for MACS. (A) Representative photographs of enzymatic fibrin gel dissolution by proteinase K. Cell-free fibrin gels (10 mg/mL) were treated with proteinase K at 2, 0.2, or 0.02 mg/mL and photographed at 25 and 90 min. A d-PBS–only control is included. Red circles indicate residual undissolved gel. (B) Representative photographs of fibrin gels after non-enzymatic urea treatment. Fibrin gels (10 mg/mL) were incubated with 5 M urea in demineralized water (Urea-H₂O) or 5 M urea in 50 mM Tris/150 mM NaCl (Urea-TrisNaCl) and photographed after 24 h. Red circles indicate intact gel persisting under both conditions and in the d-PBS control. (C) Quantification of the effect of proteinase K treatment on downstream MidiMACS sorting. DM cells (1 × 10⁶ input per group) were subjected to MidiMACS sorting after treatment with proteinase K (2 mg/mL) or left untreated. (i) Live cell recovery after MidiMACS sorting, expressed as the percentage of viable cells recovered relative to input (trypan blue exclusion) . (ii) MACS capture efficiency of CD146⁺ cells, expressed as the percentage of labelled cells retained during sorting. Data are presented as mean ± SD (n = 2 per group).

Releasing cells from the 3D fibrin matrix is a prerequisite for post-culture cell sorting, and therefore two dissolution strategies were tested fibrin gel without cells (Fig. 3) . First, proteinase K at 2 mg/mL dissolved the fibrin gel within 25 min; lower concentrations (0.2 and 0.02 mg/mL) showed progressively slower dissolution with residual gel at 90 min (Fig. 3A) . Second, urea treatment (5 M urea in water or in 50 mM Tris/150 mM NaCl) resulted in visible shrinkage and partial degradation, however, residual gel fragments persisted after 24 h (Fig. 3B) .

To test the complete gel-dissolution–to–MACS workflow, the combination of 2 mg/mL proteinase K (as the most effective dissolution condition) with MidiMACS (as the superior separation system) was selected. 2D cultured DM cells (1 × 10^6)^ were treated with 2 mg/mL proteinase K or left untreated, then processed through MidiMACS. Live cell recovery remained high in both conditions (∼80–95%) , with a slight decrease after proteinase K treatment (Fig. 3Ci) . Results showed however that the CD146^+^ capture fraction was markedly affected: controls yielded ∼55–60% capture, whereas proteinase K-treated cells dropped the result to a near 0% capture (Fig. 3Cii) . While Proteinase K effectively dissolved fibrin it (undesirably) degraded the CD146 epitope, rendering the sequential gel-dissolution–to–MACS workflow ineffective in a manner as it was anticipated.

### Spatial transcriptomics: physical processing feasible but RNA critically degraded

**Fig. 4:**
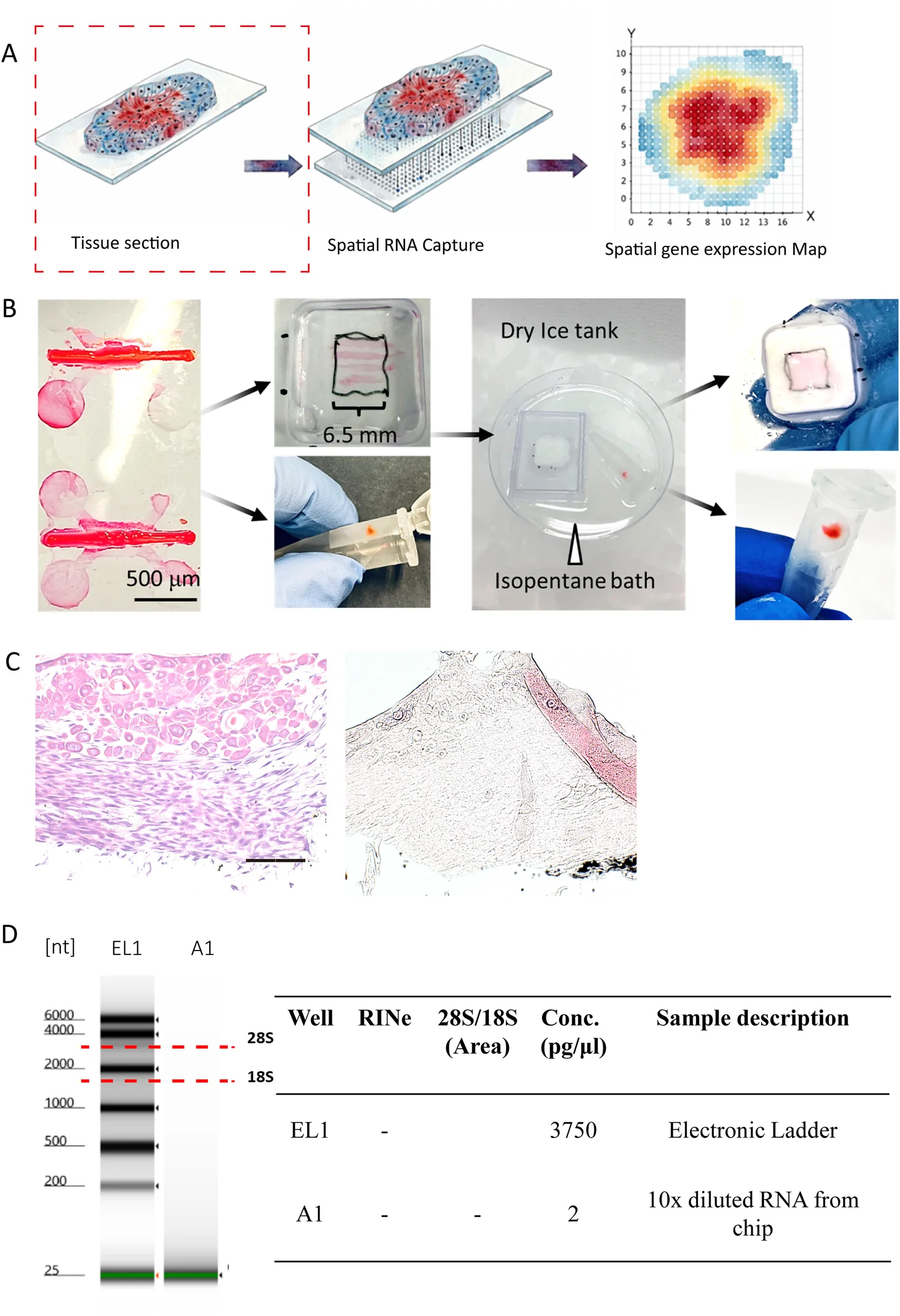
The tooth-on-chip is physically compatible with spatial transcriptomics processing but RNA is critically degraded. (A) Schematic overview of spatial transcriptomics, in which mRNA from tissue cryosections is captured by positionally barcoded arrays to generate spatially resolved gene expression maps. The compatibility of the standard upstream sample-preparation workflow for spatial transcriptomics with soft fibrin-based chip constructs was evaluated (dashed box) . (B) Sample preparation workflow. tooth-on-chip constructs were harvested and briefly stained with eosin for visualization (left) . Four constructs were aligned in a 6.5 × 6.5 mm area in OCT cryomold for cryosectioning (upper middle) , while two additional constructs were embedded separately in OCT within Eppendorf tubes as matched RNA quality controls (lower middle) . All samples were snap-frozen in an isopentane bath on dry ice (center) . Upper right: frozen OCT block. Lower right: OCT-embedded samples frozen in Eppendorf tubes for RNA quality assessment. (C) Representative images of H&E staining and destaining of tooth-on-chip cryosections. H&E staining reveals well-preserved cellular and architectural morphology within the fibrin matrix (Left) . Destaining confirms effective removal of haematoxylin and eosin signals (Right) . Partial tissue loss at section edges and localized folding artefacts reflect the mechanical fragility of the hydrogel matrix. Scale bar: 50 µm. (D) RNA quality assessment of OCT-embedded constructs processed through the fixation–embedding pipeline. Left: Bioanalyzer electropherogram comparing the processed sample (A1) with the electronic ladder (EL1) ; red dashed lines indicate the expected positions of 28S and 18S rRNA bands, neither of which is detectable in A1. Right: summary table showing undetectable RIN^e,^ absent 28S/18S ratio, and an RNA concentration of only 2 pg/µL (at 10 × dilution).

Spatial transcriptomics maps gene expression to specific locations within intact tissues, preserving spatial context (Fig. 4A) . To assess the feasibility of adapting the tooth-on-chip construct to a spatial transcriptomics workflow, constructs underwent the full upstream processing pipeline of fixation, embedding within a 6.5 × 6.5 mm capture area, cryosectioning, H&E staining, destaining, and RNA quality assessment, prior to library construction (Fig. 4B) .

H&E-stained cryosections demonstrated well-preserved tissue morphology and staining quality (Fig. 4C) . The DE and DM compartments were clearly distinguishable, with a well-defined interface maintained across sections. Hematoxylin produced distinct basophilic nuclear profiles, while eosin provided appropriate cytoplasmic contrast. Destaining was equally effective (Fig. 4C) : sections remained firmly adhered throughout the procedure, hematoxylin and eosin signals were efficiently cleared, and no reagent-induced detachment or structural disruption was observed. Together, these results confirmed that methanol fixation, cryosectioning, H&E staining, and destaining were physically compatible with hydrogel-based tooth-on-chip constructs. Nonetheless a subset of sections exhibited partial tissue loss at the edges, as well as localized folding and curling artefacts that persisted. These features are likely attributable to the mechanical fragility of the fibrin hydrogel matrix and may reduce spatial fidelity during transcript capture.

Beyond physical processing, we assessed RNA integrity to determine molecular compatibility with the spatial transcriptomics workflow. RNA quality assessment of processed sections revealed near-complete degradation (Fig. 4D) : the RIN^e^ was undetectable (reported as "–") , no measurable 28S/18S ratio was obtained, and RNA concentration was only 2 pg/µL. For reference, bulk RNA extraction from the same construct type yielded RINe values of 9.7–9.8 and concentrations three orders of magnitude higher (Fig. 1C) , confirming that RNA integrity is not intrinsically compromised within the construct.

### Raman spectroscopy and TEM detects early mineralization at the DE–DM interface

**Fig. 5:**
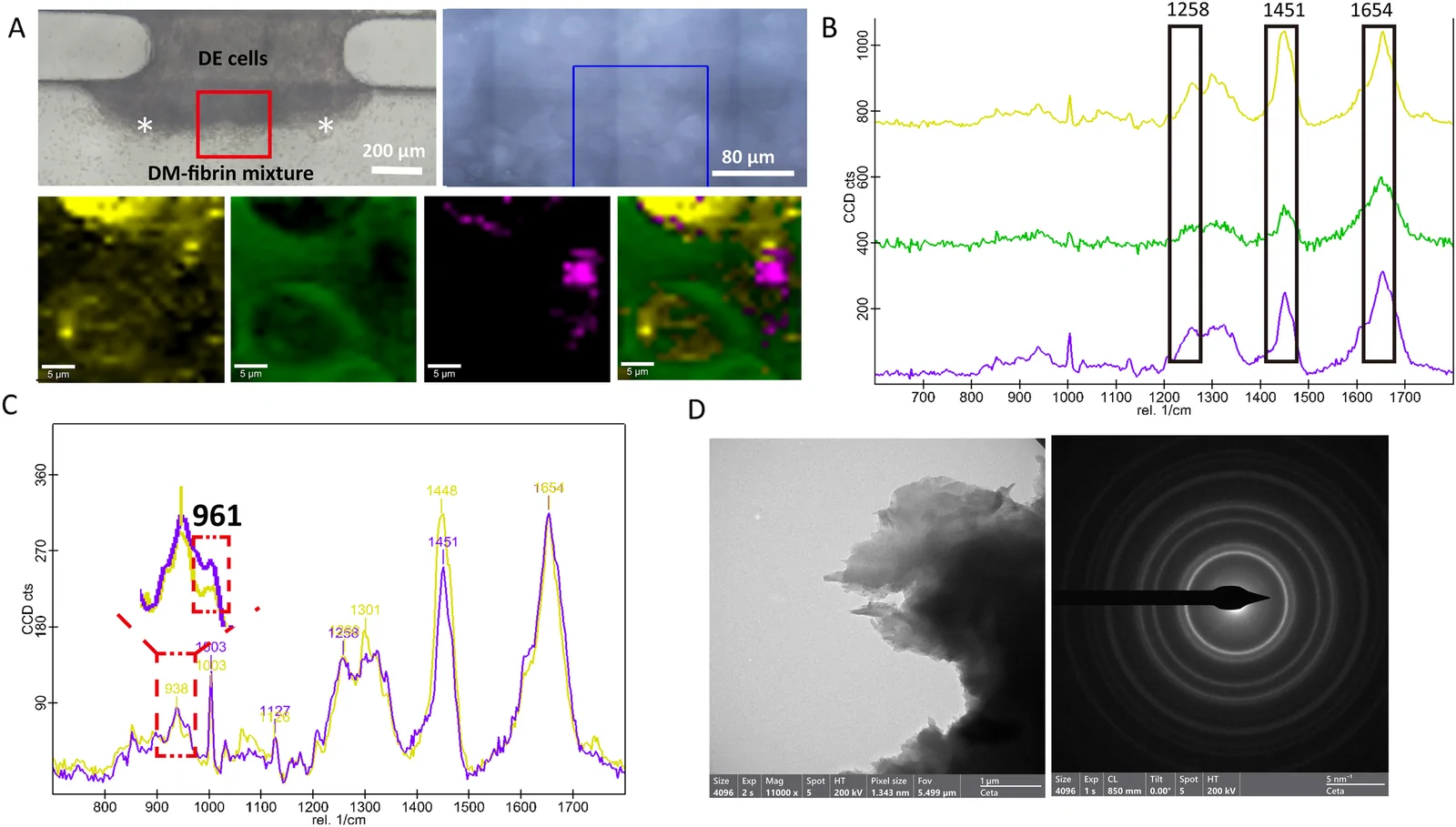
Raman spectroscopy and TEM reveal early-stage calcium phosphate deposition in 21-day tooth-on-chip constructs. (A) Raman imaging of the DE–DM interface region. A brightfield overview of the tooth-on-chip construct shows the upper channel containing DE cells and the lower channel containing the DM–fibrin mixture. The interface between the two compartments is defined by the inter-pillar region (asterisks) . The region selected for Raman scanning is indicated by the red box, and higher magnification reveals two scan areas at the interface (red and blue boxes) . Multivariate decomposition of the Raman scan data resolves three spectral components (lower panel) , visualized as individual spatial distribution maps (yellow, green, and purple/magenta) and as a merged overlay. The green component is distributed throughout the construct, consistent with the bulk fibrin matrix, whereas the yellow and purple components are spatially restricted to discrete cell-associated regions. (B) Full Raman spectra of the three spectral components. Spectra are shown over the 400–1900 cm⁻¹ range for the yellow, green, and purple components. Black dashed boxes indicate shared organic vibrational bands, including amide III (∼1258 cm⁻¹) , methylene (CH₂) bending (∼1451 cm⁻¹) , and amide I (∼1654 cm⁻¹) . (C) Expanded spectral comparison highlighting mineral-associated features. The yellow and purple spectra are shown over the 700–1800 cm⁻¹ range. A peak at 961 cm⁻¹ is observed in the purple component (red dashed box) , corresponding to the ν₁ PO₄³⁻ symmetric stretching vibration of crystalline calcium phosphate. (D) TEM characterization of DE–DM interface. The TEM micrograph (200 kV, 11,000 ×) shows a high-contrast, electron-dense region (Right) . The corresponding selected area electron diffraction (SAED) pattern exhibits multiple concentric diffraction rings, reflecting scattering from numerous randomly oriented crystalline domains within the selected area.

To evaluate functional mineralization, Raman spectroscopy was applied to map the molecular composition of 21-day constructs at the DE–DM interface (Fig. 5A, upper panels) . Multivariate spectral decomposition resolved three main spectral components, displayed in green, yellow, and purple (Fig. 5A, lower panels) . Full spectral profiles revealed shared organic peaks across all three components, including Amide III (∼1, 258 cm⁻¹) , CH₂ bending (∼1, 451 cm⁻¹) , and Amide I (∼1, 654 cm⁻¹) (Fig. 5B, black boxes) . The green component was broadly distributed throughout the construct and exhibited all of the organic signals, consistent with the bulk fibrin hydrogel matrix.

The yellow and purple components were more spatially restricted and co-localised at discrete regions of the construct, suggesting association with cell-occupied or pericellular zones where active matrix remodeling would be expected. Despite their broadly similar spectral profiles, subtle differences were observed in the 900–1000 cm⁻¹ region (Fig. 5C) : the purple spectrum displayed a sharper and more prominent peak near 939–961 cm⁻¹, whereas the yellow spectrum showed a lower and broader feature in the same region, indicating compositional heterogeneity between these two cell-associated components.

Focused inspection of this region identified a small but discernible peak at 961 cm⁻¹ in the purple spectrum (Fig. 5C, red box) . This wavenumber corresponds to the ν₁ PO₄³⁻ symmetric stretching vibration, the principal Raman marker of crystalline calcium phosphate phases including hydroxyapatite. Nevertheless, its presence at the expected wavenumber, localized within cell-associated regions, provides tentative spectroscopic evidence of early-stage calcium phosphate deposition following 21 days of osteogenic induction.

Brightfield TEM imaging of 21-day tooth-on-chip constructs revealed discrete electron-dense deposits intimately associated with an organic-rich extracellular matrix (Fig. 5D) . The deposits presented as irregularly shaped granular aggregates with diffuse boundaries and lamellar extensions, lacking the elongated crystallite morphology characteristic of mature hydroxyapatite. SAED acquired from these regions showed broad, continuous concentric rings on a diffuse background, indicative of a phase with very limited long-range crystallographic order. These ultrastructural and diffraction features were consistent with an amorphous or poorly ordered calcium phosphate precursor phase, an interpretation further supported by the weak, broad 961 cm⁻¹ phosphate peak observed in Raman spectroscopy. Together, the convergent TEM and Raman evidence indicated that calcium phosphate deposition had initiated within the matrix at 21 days, yet without clear evidence of transition to well-ordered hydroxyapatite at this time point.

## Limitations

This workflow was developed for fibrin-based tooth-on-chip constructs and should be used within defined technical and interpretive boundaries. Within this scope, the purpose was not to demonstrate how to optimize every workflow, but to determine where established methods cease to be fit for purpose when transferred to microscale, hydrogel-based systems. As these limits arise at different stages of the workflow, the methodological consequences differ across interpretation, validation, workflow design, and evidential requirements (Table 1) .

**Table 1:** Fit-for-purpose boundaries and decision pathways for tooth-on-chip analytical workflows.

	Analytical aim	Approach evaluated	Fit-for-purpose Limitation	Methodological implication
1st	Construct-level transcriptional profiling, such as treatment-versus-control comparisons	Bulk RNA extraction of intact constructs	**Interpretive boundary:** Bulk RNA yields high-integrity; Additional RNA purity assessment is required before sequence; The population-averaged signal cannot be attributed to DE or DM origin [18]	• Restrict findings as pooled DE–DM responses. • To estimate the relative contributions of DE and DM to transcriptional changes, reference-based deconvolution could be applied with suitable reference profiles, such as published dental single-cell atlases [18,19] or chip-matched profiles from DE-only and DM-only chip model [20]
2nd	Assessment of DE/DM enrichment after MACS	Morphology-based classification of the recovered MACS fractions	**Validation boundary:** Morphology suggested DE/DM enrichment, but DE cell cluster entrapment [21,22] and epithelial–mesenchymal phenotypic changes can bias visual classification [23,24]	• Validate the input, retained, and flow-through fractions using quantitative DE/DM marker panels by immunofluorescence or qPCR, with morphology retained only as supporting evidence.
3rd	Cell recovery from hydrogel for DE/DM separation	Proteinase K release → CD146-based MACS	**Workflow-compatibility boundary:** Efficient fibrin digestion compromises the CD146 surface epitope required for antibody-based capture [25], resulting an incompatibility between sample release and downstream MACS	• Redesign the release step with a candidate fibrinolytic agent, such as nattokinase [26].validated for epitope preservation, or use a matrix permitting non-destructive cell recovery. • Replace fibrin with a hydrogel supports physical recovery, such as (thermoresponsive [27,28] or photo-degradable hydrogels [29].
4th	Spatial transcriptomics of mature mineralizing constructs	Aldehyde fixation → poly (A) -dependent capture	**Workflow-compatibility boundary:** RNA fragmentation [30] and reduced transcript accessibility fall below the input threshold	• Match sample preparation to transcript-capture chemistry: prioritize fixation-free preparation [31] for poly (A) capture, or fixation-compatible probe-based platforms when fixation is required[32].
5th	Identification of the mineral phase	Raman spectroscopy or TEM	**Evidence boundary:** Mineral formation can be detected, but precise phase assignment remains limited in matrix-rich, heterogeneous deposits	• Report calcium phosphate formation conservatively • Claim apatite formation only with additional crystallographic evidence, such as micro-/synchrotron XRD, indexed SAED and HRTEM lattice-spacing analysis

These limitations differ in their methodological consequences. The first and last limitations in this table defined interpretive boundaries rather than procedural failures, i.e. (1) . Bulk RNA-seq can identify global gene-expression changes across the intact construct, and Raman/SAED can indicate the presence of calcium phosphate deposition. However, the level of interpretive resolution remains limited: bulk RNA-seq cannot localize transcriptomic changes to DE or DM compartments, and (2) Raman/SAED alone cannot definitively identify the mineral phase.

The 2nd limitation is on the level of the protocol: MACS enrichment may remain suitable, but morphology is not an appropriate primary metric for evaluating DE/DM purity in this system. Accordingly, morphology should be retained only as a supportive readout. Fit-for-purpose use of the sorted fractions requires quantitative molecular validation, preferably at the cell level by quantitative immunofluorescence, with qPCR serving as complementary population-level confirmation.

The 3rd and 4th limitations can be more seen as incompatibilities on the platform-level: the upstream preparation step compromises the molecular feature required by the downstream assay, thus making downstream parameter optimization insufficient to restore performance. In these cases, the workflow must be modified at the level of sample release, fixation strategy, or downstream platform selection. Thus, these practical contributions are therefore not to validate every potential workflow, but to identify where compatibility fails, distinguish sample-related limitations from processing-related limitations, and define the decision points and acceptance criteria required for subsequent implementation.

Taking these limitations all together, also defines how the resulting data should be reported. Transcriptomic changes measured from intact constructs should be presented as construct-level observations unless cellular origin is resolved by physical separation, spatial profiling, or validated deconvolution. Mineralization should be reported as calcium phosphate formation, with apatite assignment reserved for cases supported by further chemical or crystallographic validation. This reporting framework then aligns the biological claims with the analytical resolution of the workflow and provides a practical standard for studies adopting this pipeline.

## CRediT author statement

Chong Huang: Contributed to conception and design, data acquisition, analysis, and interpretation, drafted the manuscript; Chloe Tijhof: Contributed to data acquisition, analysis, and interpretation; Wei Ji: Contributed to conception, data interpretation, and critically revised the manuscript; Fang Yang: Contributed to conception and design, data acquisition, analysis, and interpretation, critically revised the manuscript; X.Frank Walboomers: Contributed to conception and design, data acquisition, analysis, and interpretation, critically revised the manuscript.

## Ethics statements

DM cells were harvested from wisdom teeth of young adult patients (18–24 years of age) in our in-house clinic.The use of human primary cells in this study was reviewed by the Medical Research Ethics Committee (METC/CMO) Arnhem-Nijmegen, Radboud University Medical Center, Nijmegen, the Netherlands (currently METC Oost-Nederland, 2020–7093) . The committee determined that the study, which used anonymized residual tissue (teeth obtained as surgical waste following third-molar extraction) , did not fall under the scope of the Dutch Medical Research Involving Human Subjects Act (WMO) .

DE cells were derived from the third molar tooth buds of 6-month-old pigs for food production, and thus did not require the use of animals specifically for experimental purposes.

## Declaration of competing interests

The authors declare that they have no known competing financial interests or personal relationships that could have appeared to influence the work reported in this paper.

## Data Availability

Data will be made available on request.

## Appendix Supplementary materials

Image, application 1
