# Supplementary material for: A multi-modal profiling workflow of transcriptomics and biomineralization demonstrated on a tooth-on-chip model
Source: MethodsX. 2026 Aug 31;17:104137. doi: 10.1016/j.mex.2026.104137 (PMC13571508; doi:10.1016/j.mex.2026.104137)
Supplement: Supplementary file 1 [file mmc1.docx]

Figure S1. Representative Agilent 2100 Bioanalyzer electropherograms of RNA isolated from intact Tooth-on-Chip constructs. Electropherograms from three representative samples (A1–C1) show the characteristic 18S and 28S rRNA peaks used for RNA integrity assessment (red box). The high-molecular-weight region is highlighted in blue, with no prominent signal indicative of substantial genomic DNA contamination. Fluorescence intensity is plotted against RNA fragment size.
